# Top–Down Proteomics of Skinned Human Muscle Fibers Reveals Proteoform‐Resolved Fiber‐to‐Fiber Variability

**DOI:** 10.1002/jms.70040

**Published:** 2026-02-22

**Authors:** Mallory C. Wilson, Zhan Gao, Justin R. Lopez, Yanlong Zhu, Szczepan S. Olszewski, Adam R. Konopka, Gary M. Diffee, Ying Ge

**Affiliations:** ^1^ Department of Chemistry University of Wisconsin‐Madison Madison Wisconsin USA; ^2^ Department of Cell and Regenerative Biology University of Wisconsin‐Madison Madison Wisconsin USA; ^3^ Department of Kinesiology University of Wisconsin‐Madison Madison Wisconsin USA; ^4^ Human Proteomics Program, School of Medicine and Public Health University of Wisconsin‐Madison Madison Wisconsin USA; ^5^ Division of Geriatrics and Gerontology, Department of Medicine University of Wisconsin‐Madison Madison Wisconsin USA; ^6^ Wisconsin Nathan Shock Center of Excellence in the Basic Biology of Aging University of Wisconsin‐Madison Madison Wisconsin USA

**Keywords:** human muscle fiber, sensitivity, single cell heterogeneity, top–down proteomics

## Abstract

Human skeletal muscle is composed of highly heterogeneous single muscle fibers (multinucleated single cells) that are commonly classified as fast or slow fiber types, yet proteoform‐resolved characterization of individual human muscle fibers remains lacking. Herein, we establish a high sensitivity top–down proteomics method for the analysis of single human muscle fibers (hSMFs). Specifically, we have optimized the surfactant‐free extraction protocol for analysis of chemically permeabilized (“skinned”) hSMFs, a common preparation used to isolate the sarcomere prior to contractile measurements. This approach enables robust and reproducible proteoform‐level coverage of key sarcomeric proteins from individual fibers using top–down LC–MS/MS. With this method, we identified extensive inter‐ and intra‐donor fiber‐to‐fiber variability in isoform expression and proteoform abundance in hSMFs extracted from the heterogeneous vastus lateralis muscles. Together, these results demonstrate the capability of single‐fiber top–down proteomics to resolve proteoform‐level heterogeneity in human skeletal muscle and establish a methodological foundation for future studies towards elucidating skeletal muscle biology and understanding muscle‐related diseases. Source data for this manuscript is available via the MassIVE repository at massive.ucsd.edu with identifier: MSV000100493.

## 1 Introduction

Human skeletal muscles are composed of diverse single muscle fiber bundles that function collectively to perform the continuum of complex maneuvers associated with bipedal movement and balance, with their function directly dependent upon relative fiber type distribution [1, 2, 3]. These single muscle fibers are large, multinucleated single cells that are classified into different types based on contractile properties and myosin heavy chain (MYH) expression, including a “slow” (Type I) and two “fast” (Types IIA and IIX) fiber types [3, 4, 5]. While MYH‐based fiber typing has been widely used to group and characterize individual muscle fibers in relatively homogeneous model systems such as rodents, this classification alone lacks the resolution needed to characterize heterogeneous human skeletal muscles, which often express a continuum of pure and hybrid fiber types [3, 5, 6]. Therefore, considerable efforts have been devoted to developing approaches that enable comprehensive molecular profiling of individual muscle fibers, including multiomic strategies [5].

Mass spectrometry (MS)‐based methods for single cell proteomics have emerged as a powerful approach for elucidating cellular heterogeneity in morphologically analogous cells, including muscle fibers [7, 8, 9, 10, 11, 12]. To date, most single cell proteomics studies rely on the bottom–up approach for global proteome analyses of single cells [5, 12, 13, 14, 15, 16, 17], which are inherently limited in their ability to resolve the proteoforms [18] arising from genetic variation, alternative splicing isoforms, and post‐translational modifications (PTMs) that play critical roles in defining functional performance [18, 19, 20]. In contrast, top–down proteomics [21, 22, 23, 24] directly analyzes intact proteoforms, enabling unbiased assessment of isoform expression and PTMs, and has increasingly been applied to integrative studies aimed at linking molecular composition with functional phenotypes [25, 26, 27, 28, 29, 30, 31, 32]. Despite these advantages, high sensitivity top–down proteomics studies at the single cell level remain challenging due to the limited protein content of a single cell and the complexity of proteoform abundance as a response to environmental and physiological factors [11, 21, 26, 27, 33, 34].

Previously, we have established an extraction protocol for the top–down analysis of single skeletal muscle fibers, which are multinucleated single cells, and revealed proteoform heterogeneity in single muscle fibers isolated from three distinct muscle types [11]. These studies were conducted in relatively homogeneous rodent muscle, whereas human skeletal muscle exhibits substantially greater fiber‐type and proteoform heterogeneity [5]. In addition, top–down proteomics was performed on the intact (unskinned) muscle fibers. In this manuscript, we adapt and refine our surfactant‐free top–down proteomics protocol for heterogenous human single muscle fibers (hSMFs) after membrane permeabilization, or “skinning” [35], a common chemical membrane permeabilization technique used to isolate the sarcomere prior to contractile measurements. We have applied this optimized method to hSMFs isolated from human vastus lateralis, a frequently studied and highly heterogeneous antigravity muscle in the human thigh [2, 31]. Using this optimized workflow, we identified pronounced inter‐ and intra‐donor fiber‐to‐fiber variability in isoform expression and proteoform abundance among hSMFs, underscoring the importance of proteoform‐resolved, single‐fiber analyses for characterizing molecular heterogeneity in human skeletal muscle.

## 2 Experimental Procedures

### 2.1 Reagents and Chemicals

All reagents were purchased from Sigma‐Aldrich Inc. unless noted otherwise. High performance liquid chromatography‐mass spectrometry (LC–MS) grade water and acetonitrile were purchased from MilliporeSigma. HALT protease and phosphatase inhibitor cocktail was purchased from Thermo Fisher Scientific.

### 2.2 hSMF Isolation

Human single muscle fibers (hSMFs) were isolated from the vastus lateralis muscles of healthy donors (25–30 yrs old) obtained using biopsy procedures under local anesthesia (1% xylocaine) approved by the Institutional Review Board (IRB) of The University of Wisconsin‐Madison Health Sciences (IRB#: 2021‐1519). The isolation procedure is as follows: Samples were cleared of visible adipose and connective tissue. Bundles of 20–50 hSMFs were dissected from the donor vastus lateralis muscles and placed in a petri dish containing relax solution (100 mM KCl, 1.75 mM EGTA, 10 mM Imidazole, 4 mM ATP, 5 mM MgCl_2_; pH 7.0) under a confocal microscope. Fiber bundles were secured to a capillary tube using 6/0 braided silk sutures on each end, then submerged in a skinning solution (2× relax solution and 50% v/v glycerol) for 24 h at −4°C. After 24 h, the fiber bundles were placed in fresh skinning solution at −20°C for storage until the day of contractile measurement.

### 2.3 Contractile Measurement Collection

Contractile measurements were collected for each fiber using a previously described workflow [36, 37]. On the day of data collection, an individual skinned fiber was removed from the end of each bundle using fine‐point tweezers and attached between a capacitance‐gauge transducer (Model 403, sensitivity of 20 mV/mg and resonant frequency 600 Hz; Aurora Scientific) and a DC torque motor (Model 308; Aurora Scientific) by securing the ends to stainless steel troughs with 6/0 braided silk sutures. The length of the preparation was adjusted so that sarcomere length was set to 2.5 μm in relaxation solution and sarcomere length was monitored in pCa 4.5 to ensure sarcomere length did not change significantly during isometric activation. Length changes during contractile measurements were introduced at one end of the preparation driven by voltage commands from a PC via a 16‐bit D/A converter. Force and length signals were digitized at 1 kHz using a 16‐bit A/D converter and stored on a PC using custom software in LabView for Windows (National Instruments Corp.). The experimental chamber contained three troughs into which the single fiber was moved to effect rapid solution changes. The apparatus was cooled to 15°C using Peltier devices (Cambion) and a circulating water bath. The entire mechanical apparatus was mounted on a pneumatic vibration isolation table having a cut‐off frequency of ~1 Hz.

Individual fibers (~3 mm in length) were removed from the apparatus and placed in a labeled low‐protein binding microcentrifuge tube. The location of the fiber in the tube was outlined with permanent marker and samples were flash‐frozen at −80°C until the day of extraction. In total, there were *n* = 6 donors. An additional two fibers were collected from donor D4 (total *n* = 3) for a triplicate intra‐donor fiber comparison.

### 2.4 Surfactant‐Free Protein Extraction

On the day of extraction, fibers were briefly thawed (~1 min) on ice. All sample manipulations were performed on ice to minimize protein degradation and artificial oxidation. To rinse any leftover relaxation solution, 40 μL of ammonium acetate were slowly dripped over each fiber with a pipette without dislodging the fiber. Fibers were briefly centrifuged (1000 ×*g*, 1 min, 4°C) and remaining ammonium was removed. Fibers were suspended in 20 μL of extraction solution (100% HFIP, 1X HALT protease and phosphatase inhibitor, and 10 mM L‐methionine), and gentle up‐and‐down pipetting was used to dislocate the fiber from the LoBind sidewall, ensuring the fiber had not stuck to the pipette tip. After the fibers visibly dissolved in solution (~1 min), samples were immediately centrifuged (21 000 ×*g*, 15 min, 4°C). After centrifugation, extracts were buffer exchanged into 0.2% FA via an Amicon 10 kDa molecular weight cutoff spin filter (MilliporeSigma) (15 000 ×*g*, 5 × 5 min, 4°C). Samples were then concentrated to a final volume of ~20 μL (15 000 ×*g*, 30 min, 4°C). Finally, 10 μL of each extract were used to estimate protein concentrations using a Bradford assay. The remaining ~10 μL were transferred to HPLC vials and placed in a NanoAcuity autosampler.

### 2.5 Liquid Chromatography and MS

OtofControl 3.4 (Bruker Daltonics) was used to collect all LC–MS data. Protein separation was performed using reverse‐phase chromatography with a NanoAcuity Ultra‐High Pressure LC system (Waters, Milford, MA, USA) similarly as previously reported [11, 38]. Injection volumes were adjusted to enable 500 ng of total protein. Elution was performed from a home‐packed C4 column (200 × 0.250 mm, 2.7 μm, 1000 Å C4 (Halo)) held at 50°C with a flow rate of 5 μL/min. Mobile phase A (MPA) contained 0.2% FA in H_2_O, and mobile phase B (MPB) contained 0.2% FA in ACN, with a 65 min RPC gradient of the following MPB concentrations: start at 10% MPB, hold 10% until 5 min, 25% at 15 min, 40% at 35 min, 50% at 45 min, 95% at 55 min, adjusted back to 10% at 55.1 min, and held at 10% until 65 min. Eluted proteins were electrosprayed into a high‐resolution Impact II quadrupole time‐of‐flight (QTOF) mass spectrometer (Bruker Daltonics, Bremen, Germany). The end plate offset was set to 500 V and the capillary voltage was set to 4500 V. The nebulizer was set to 0.5 bar with a dry gas flow rate of 4.0 L/min at 200°C. Mass spectra were collected at a scan rate of 1 Hz over 300–3000 m/z with a quadrupole low mass set to 650 m/z.

### 2.6 Data Analysis

LC–MS data was processed and analyzed using DataAnalysis (v4.3, Bruker Daltonics) software [32]. Maximum Entropy Deconvolution of spectra was performed with a resolving power of 60 000 for isotopically resolved proteins. The Top 5–7 most abundant charge state ions from the non‐deconvoluted spectra were used to produce extracted ion chromatograms (EICs), and relative abundances of isoforms were measured using the ratios of the area under the curve (AUC) of each isoform's EIC. Relative quantification of proteoforms was performed by deconvoluting mass spectra using the Maximum Entropy Deconvolution algorithm and taking the ratio of the highest peak intensity of the proteoform to the summed intensities of all proteoforms of that protein. Total phosphorylation of proteins with multiple phosphorylation sites was calculated as the ratio of the sum of the peak intensity of all phosphorylated proteoforms (multiplied by the integer number of phosphorylated sites on that proteoform) to the sum of all proteoform peak intensities of that protein, similarly as reported previously [11, 25, 30, 38]. The sophisticated numerical annotation procedure (SNAP) algorithm was applied to determine the monoisotopic masses of all observed ions.

## 3 Results and Discussion

### 3.1 High‐Sensitivity Top–Down Proteomics Method for Skinned hSMFs

We previously established a high‐sensitivity, surfactant‐free extraction protocol using hexafluoroisopropanol (HFIP) for top–down analysis of single skeletal muscle fibers, enabling the detection of proteoform heterogeneity among distinct muscle types collected from the rodents [11]. Though successful with intact, non‐skinned rodent muscle fibers, the original extraction using 25% HFIP was unable to reproducibly extract protein from skinned hSMFs following treatment for contractile functional measurement. This is likely attributable to the skinning process, which is a chemical membrane permeabilization that disrupts or removes the sarcolemma while preserving the internal sarcomeric lattice for controlled contractile measurements [39, 40, 41]. This dramatic change in sarcolemmal permeability would induce structural differences, thereby altering the physical constraints that otherwise facilitate effective solubilization with lower concentrations of HFIP [39]. We therefore hypothesized that increasing HFIP concentration could allow effective surfactant‐free disruption of the exposed sarcomere, as higher concentrations of HFIP have been shown to provide comparable or improved solubilization in intact protein extractions without artificially affecting proteoform abundance [38]. Indeed, when the concentration was increased to 100% HFIP, we achieved successful sarcomere lysis from skinned hSMFs (Supplementary Figure S1) [38]. Each hSMF was completely solubilized without any mechanical homogenization, as evident by visualization of the complete dissolution of hSMFs in the solution. This eliminated the need for the freeze–thaw lysis steps described in the original protocol developed for non‐skinned muscle fibers [11].

**FIGURE 1:**
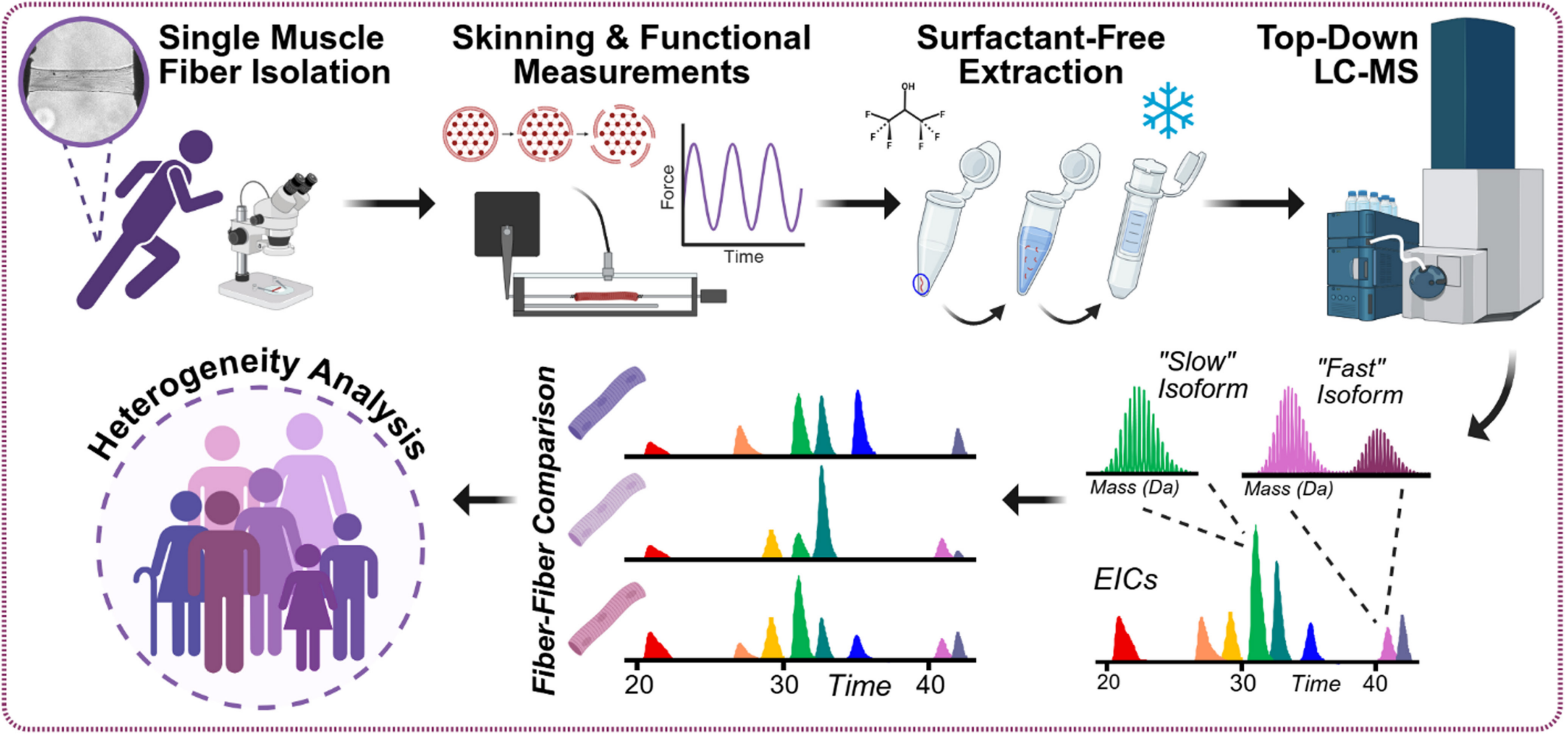
Experimental workflow overview. Human single muscle fibers (hSMFs) are isolated from donor vastus lateralis muscles. Following chemical membrane permeabilization (skinning) and functional assessment, sarcomeric proteins are extracted from individual fibers using a 100% hexafluoroisopropanol (HFIP) solution. Subsequent top–down LC–MS analysis achieved proteoform‐resolved characterization of hSMFs, enabling unbiased analyses for assessing intra‐ and inter‐donor hSMFs heterogeneity.

**FIGURE 2:**
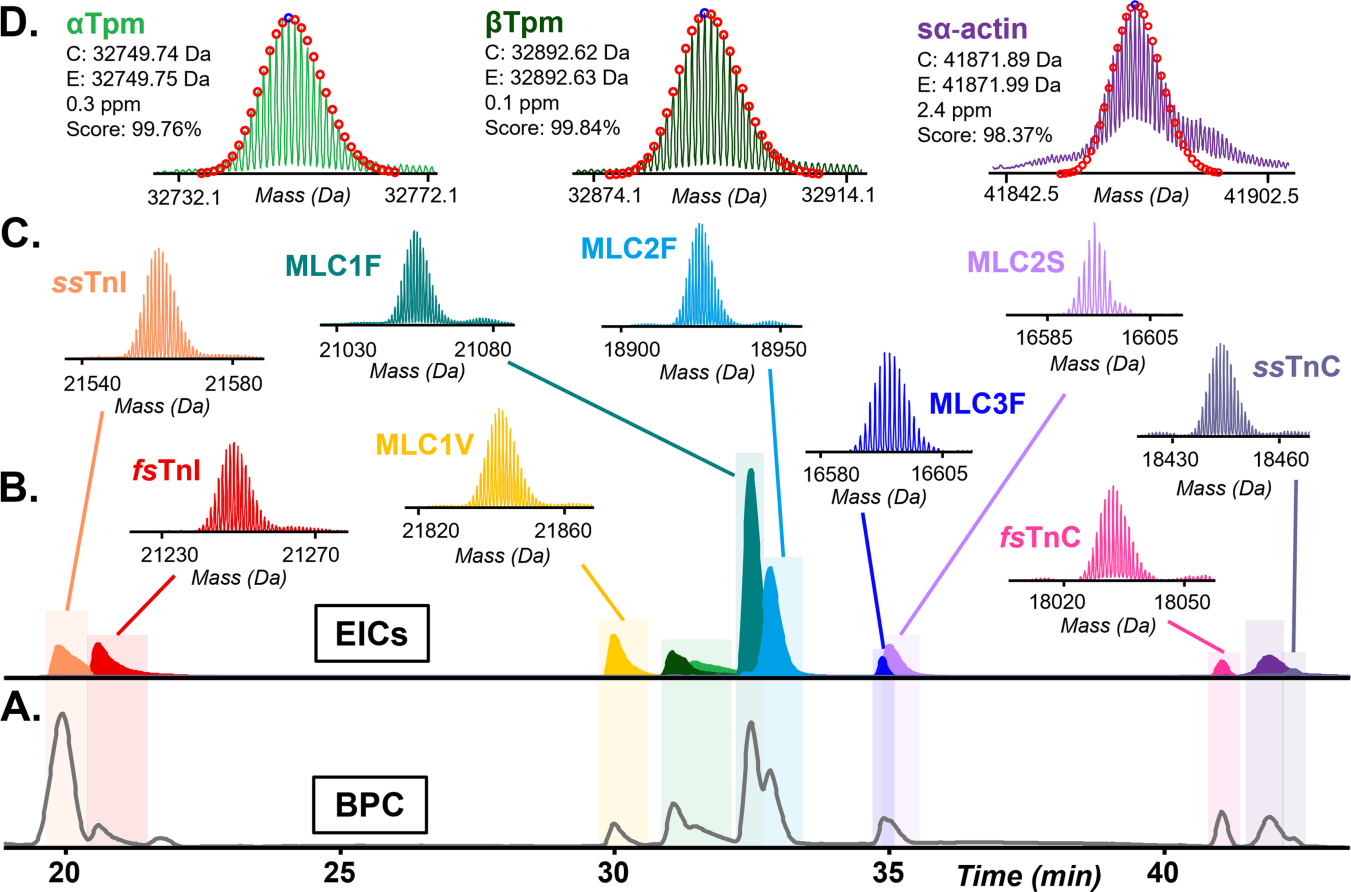
Top–down proteomics analysis of sarcomere proteins extracted from a hSMF. (A) Base peak chromatogram (BPC) and (B) extracted ion chromatogram (EIC) illustrating the separation of sarcomeric proteins. (C) Representative deconvoluted mass spectra of fast and slow sarcomeric protein isoforms, including troponin T (cTnT), slow‐ and fast‐skeletal troponin I (*ss*TnI, *fs*TnI), slow and fast myosin light chain 1 (MLC1V, MLC1F), slow and fast myosin light chain 2 (MLC2S, MLC2F), fast myosin light chain 3 (MLC3F), and fast and slow troponin C (*ss*TnC, *fs*TnC). (D) Isotopic fitting of tropomyosin isoforms and sα‐actin enabled by MASH Native, showing calculated mass (C), experimental mass (E), error in parts per million (ppm), and the Gaussian isotopic distributions (in red dots) on the deconvoluted spectra with corresponding curve fit‐score (%).

With these modifications, we achieved robust and reproducible top–down LC–MS proteomics analysis of individual hSMFs following functional treatment and assessment, enabling relative fast and slow isoform expression and proteoform abundance quantification for heterogeneity analyses of sarcomere proteins in hSMFs (Figure 1). We identified key sarcomeric proteins at the MS1 level in individual hSMFs: slow‐skeletal troponin I (*ss*TnI, *TNNI1*), fast‐skeletal troponin I (*fs*TnI, *TNNI2*), slow isoform myosin light chain 1 (MLC1V, *MYL3*), alpha‐tropomyosin (αTpm, *TPM1*), beta‐tropomyosin (βTpm, *TPM2*), fast isoform myosin light chain 1 (MLC1F, *MYL1*), fast isoform myosin light chain 2 (MLC2F, *MYL11*), fast isoform myosin light chain isoform 3 (MLC3F, *MYL1*), slow isoform myosin light chain 2 (MLC2S, *MYL2*), fast‐skeletal troponin C (*fs*TnC, *TNNC2*), sarcomeric alpha‐actin (sα‐actin, *ACTA1*), and slow‐skeletal troponin C (*ss*TnC, *TNNC1*) (Table S1, Figure 2A–D). Instrument performance and reproducibility were demonstrated by consistent retention times and spectral intensities of technical replicates, with three highly reproducible injection replicates from the same hSMF within the instrument's linear response range (Supplementary Figures S2, S3). Additionally, no evidence of sarcomeric protein degradation or artifactual oxidation was observed, indicating compatibility of the optimized extraction with skinned fiber preparations, which results in modest morphological changes but otherwise similar function to non‐skinned fibers [39, 40]. Though we do not perform quantitative biological comparisons in this manuscript, this workflow is fully compatible with label‐free top–down proteomic analyses described previously [11, 25, 30, 38, 42].

### 3.2 Intra‐Donor Fiber‐to‐Fiber Variability in hSMF Revealed by Top–Down Proteomics

**FIGURE 3:**
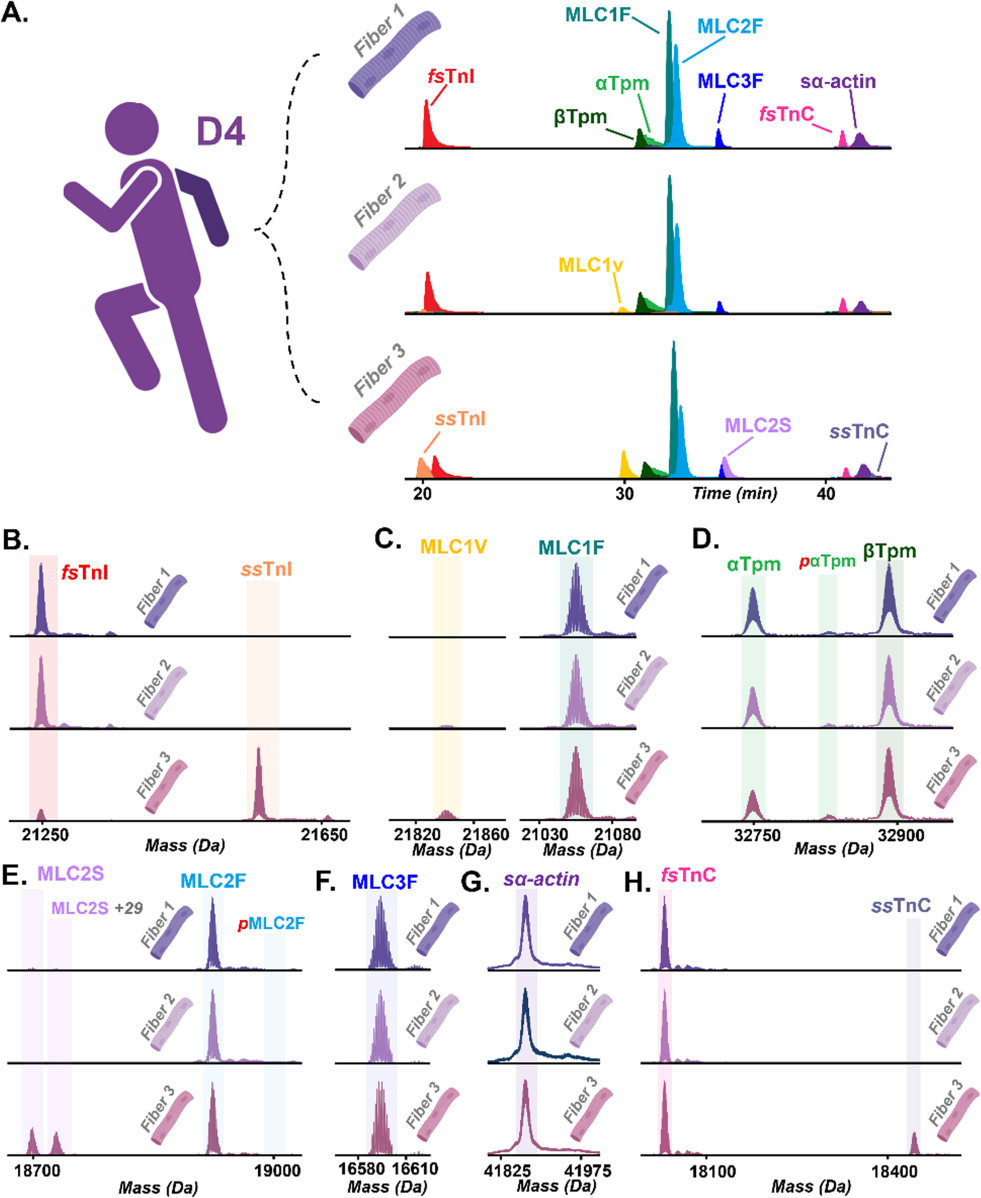
Fiber‐to‐fiber variability in isoform and proteoform abundance in hSMFs from a single donor. (A) EICs of a triplicate of fibers from a triplicate of hSMFs isolated from the same donor (D4), displaying the relative abundance of *fs*TnI, ssTnI, MLC1V, βTpm αTpm, MLC1F, MLC2F, MLC3F, MLC2S, *fs*TnC, sα‐actin, and *ss*TnC. Deconvoluted mass spectra were averaged across isoform‐specific elution time windows to assess relative proteoform abundance of (B) *fs*TnI and *ss*TnI, (C) MLC1V and MLC1F, (D) βTpm and αTpm, (E) MLC2F and MLC2S, (F) MLC3F, (G) sα‐actin, and (H) *fs*TnC and *ss*TnC.

The human vastus lateralis muscle is well‐established as a heterogeneous muscle, providing an appropriate within‐donor comparison to evaluate the sensitivity of the method for detecting fiber‐to‐fiber variability, an important limitation in biological and disease studies that rely on pooled fibers or single‐donor averages [5]. To demonstrate the efficacy of our optimized method, we performed our extraction on a triplicate of hSMFs from a single donor's (donor ID: D4) vastus lateralis muscles (*n* = 3 fibers) after skinning and subsequent standard functional measurement collection procedures (see Experimental Procedures). EICs, or a trace of ion(s) of interest eluting from the HPLC column as a function of time, exhibited highly consistent elution times of sarcomeric proteins from all three fibers, confirming reproducible extraction efficiency across samples despite the heterogenous nature of isoform expression (Figure 3A). All three fibers expressed isoforms *fs*TnI, βTpm, αTpm, MLC1F, MLC2F, MLC3F, *fs*TnC, and sα‐actin. However, the relative abundances of each fast isoform were reduced in hSMFs when their corresponding slow isoform was present. Specifically, slow isoforms MLC1V and *ss*TnI were detected in Fiber 3 and—at much lower abundances—in Fiber 2. MLC2S was detected in both Fiber 1 and Fiber 3 but was present at a considerably lower to almost undetectable abundance in Fiber 1. Only Fiber 3 is presented with detectable *ss*TnC. Moreover, we detected variability in the relative abundances of myosin light chains (MLCs), similar to the homogenous rodent muscle study (Figure 3A,C,E,F) [11]. Specifically, the ratio of MLC3F to MLC1F ranged from 0.05 to 0.11 across the triplicate fibers (Figure 3A). MLCs bind to and stabilize the lever‐arm region of MYH isoforms and are known to modulate cross‐bridge kinetics and contractile function, such that heterogeneity in MLC composition can contribute to fiber‐specific mechanical properties [2].

Additionally, this method enabled the assessment of the PTMs of each isoform, including common proteoforms such as the monophosphorylated forms of αTpm and MLC2. Deconvoluted spectra of each isoform were used to further characterize the hSMFs and identify potential fiber‐to‐fiber differences in the sarcomeric proteoforms (Figure 3B–H). In particular, the hSMF replicates had total α‐Tpm phosphorylation values ranging from 0.11 to 0.18 (Figure 3D). Importantly, this degree of heterogeneity would be obscured in bulk or pooled analyses. The extensive fiber‐to‐fiber variation observed within a single donor underscores the unique advantage of single‐fiber top–down proteomics after membrane permeabilization for mechanical measurement collection, particularly for studies of heterogeneous human muscle where functional and molecular measurements obtained from different fiber cohorts cannot be directly reconciled.

### 3.3 Top–Down Proteomics Reveals Fiber‐to‐Fiber Variation in hSMFs From Different Donors

**FIGURE 4:**
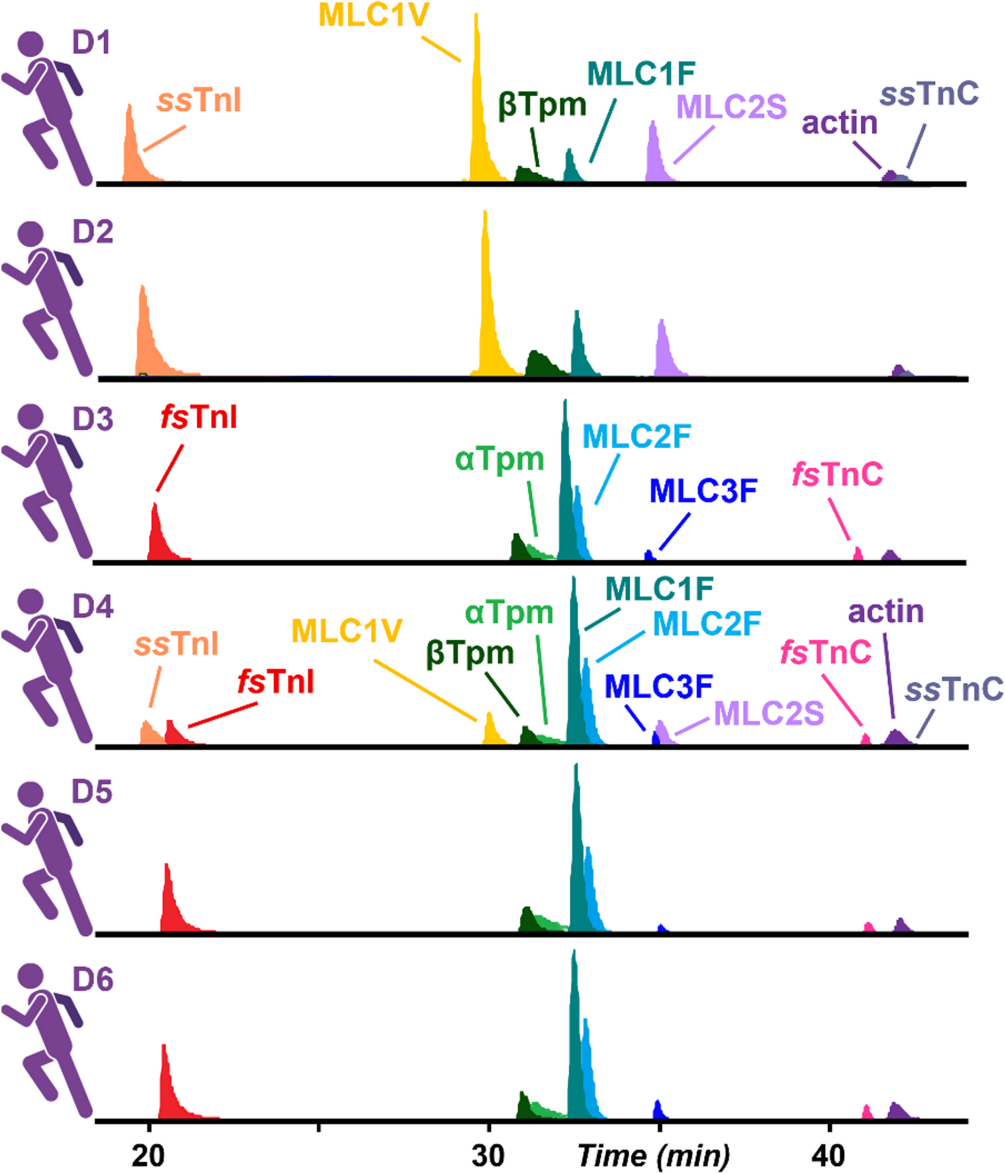
Inter‐donor heterogeneity of isoform expression in hSMFs. Extracted ion chromatograms (EICs) of detected fast and slow isoforms from multiple donors, showing consistent sarcomere protein elution times with pronounced fiber‐to‐fiber heterogeneity in relative abundance between slow and fast isoforms.

**FIGURE 5:**
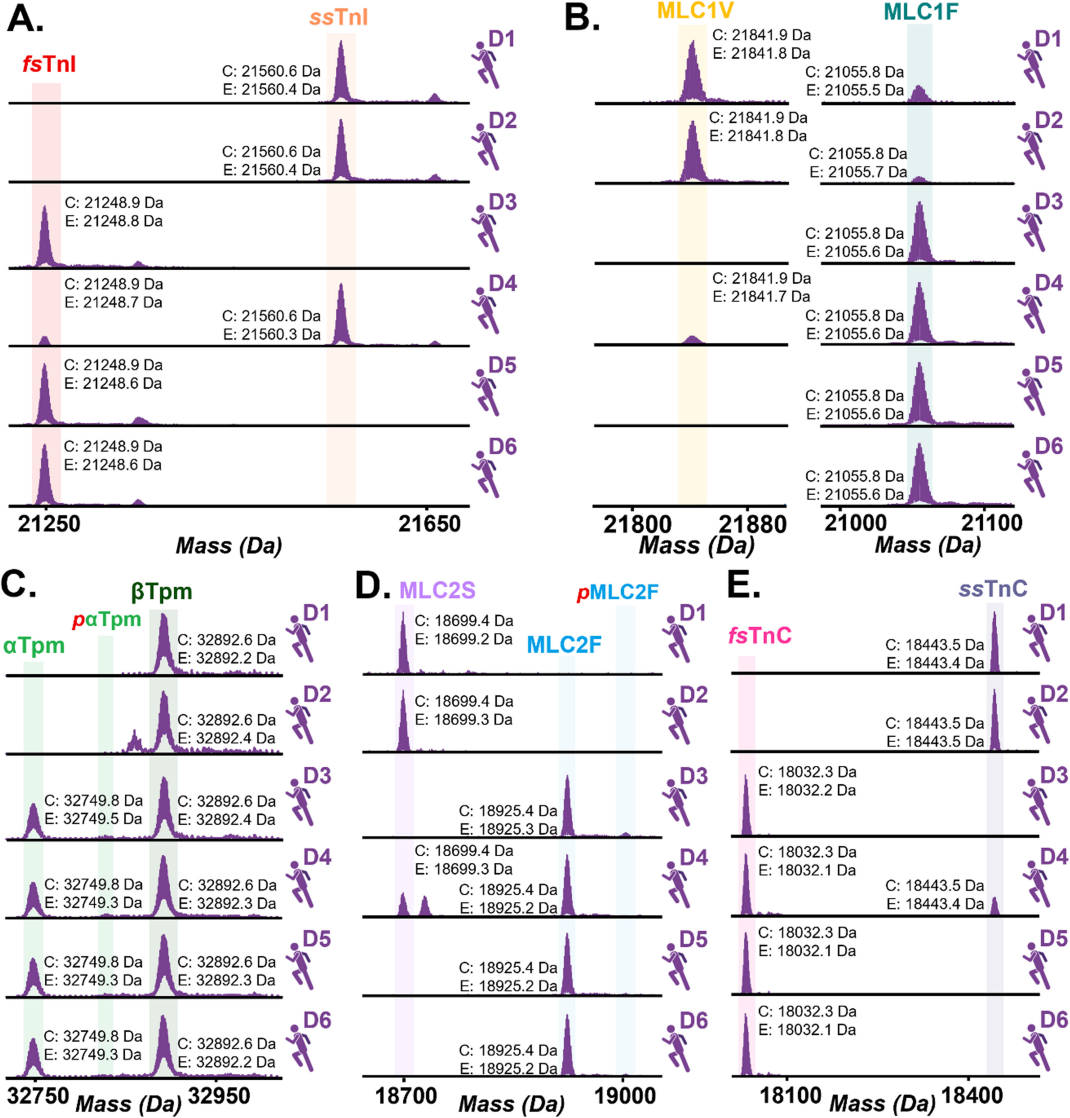
Proteoform‐resolved fiber‐to‐fiber variability in all donor hSMFs. Deconvoluted spectra of fast and slow isoform pairs displaying relative abundance of all proteoforms, including (A) *fs*TnI and *ss*TnI, (B) MLC1V and MLC1F, (C) αTpm and βTpm, (D) MLC2S and MLC2F, and (E) *fs*TnC and *ss*TnC. A calculated (C) and an experimental (E) most abundant masses are reported.

Next, we sought to evaluate the proteoform‐resolved heterogeneity of hSMFs obtained from multiple donors. Accordingly, proteins were extracted from skinned hSMFs from donor vastus lateralis muscles (*n* = 6 donors, 1 fiber per donor) following skinning and functional measurement procedures (see Experimental Procedures). The base peak chromatograms (BPCs) exhibit highly consistent elution times, confirming reproducible extraction efficiency across samples despite the pronounced variability in isoform expression (Supplementary Figure S4). EICs of all proteins within each sample further demonstrated consistent elution times and separation of fast and slow sarcomere proteins in the hSMFs (Figure 4). All hSMFs in the multi‐donor cohort expressed fast and slow sarcomeric isoforms, with the relative abundances varying considerably between donors, and were more heterogeneous than in the intra‐donor fiber comparison. Two donors' hSMFs (D1, D2) nearly exclusively expressed the isoforms of each protein that are associated with slow‐twitch muscle fibers, including *ss*TnI, MLC1V, MLC2S, and *ss*TnC. In stark contrast, three other donors (D3, D5, D6) predominantly expressed sarcomeric isoforms associated with fast‐twitch fibers, including *fs*TnI, αTpm, MLC2F, MLC3F, and *fs*TnC. Only a single fiber taken from the D4 donor triplicate expressed a balanced mix of all fast and slow isoforms. The fast isoform MLC1F was present in all samples, though its abundance was decreased in fibers primarily composed of slow isoforms. βTpm and sα‐actin were consistently present in all fibers. Variability in the ratio of MLC3F to MLC1F was observed in this extended cohort as well. In addition to isoform composition, this workflow enabled the assessment of the PTMs at the proteoform level, with the total phosphorylation of αTpm ranging from 0.06 to 0.11 across all hSMFs (Figure 5A–E, Supplementary Figures S5, S6). Notably, a potential polymorphism of MLC2S (+29 Da mass shift) was exclusively detected in the donor D4 fibers, further demonstrating the sensitivity of the method for resolving subtle proteoform differences at the single‐fiber level (Figures 3E, 5D).

Given the limited number of fibers analyzed, this study is not designed to support definitive biological conclusions for either intra‐ or inter‐donor comparisons. Instead, these qualitative observations are consistent with previous reports indicating that muscle fiber physiology reflects a complex, fiber‐specific combination of isoforms and proteoforms that cannot be fully described by functional measurements alone [5]. Without utilizing this optimized protocol, the extent of myofilament proteoform heterogeneity observed at the single fiber level across both intra‐ and inter‐donor comparisons would be obscured in bulk or pooled analyses. These findings underscore the necessity of single‐fiber top–down proteomics for characterizing proteoform‐level heterogeneity in human skeletal muscle and expand upon the field's growing knowledge of the diverse fiber type continuum.

## 4 Conclusions

In this study, we have established a surfactant‐free protein extraction workflow to enable high sensitivity top–down proteomics of human single muscle fibers following membrane permeabilization and mechanical function assessment. This approach allows proteoform‐resolved characterization of individual fibers, revealing substantial fiber‐to‐fiber heterogeneity in isoform expression and proteoform abundance in the vastus lateralis muscle within the same donor. Application of this workflow to single muscle fibers from multiple donors further revealed pronounced inter‐donor variability, underscoring the ability of top–down proteomics to resolve even subtle proteoform differences at the single‐fiber level. These results also highlight the importance of collecting both functional and proteomic data from the same fiber rather than performing parallel analyses of separate fiber cohorts, which could bias or obscure the subtle differences observed in this study. Together, this optimized protocol provides a sensitive and accessible analytical platform for future quantitative studies of single human muscle fiber heterogeneity in biological, disease, preclinical, and clinical studies.

## Funding

This work was supported by the National Institutes of Health, HL109810, GM117058, S10 OD018475; National Science Foundation, 2137424.

## Conflicts of Interest

The authors declare no conflicts of interest.

## Supporting information


**Table S1:** Summary of identified skeletal sarcomere proteins.
**Figure S1:** Method optimization of surfactant‐free extraction of proteins from skinned human single muscle fibers (hSMFs).
**Figure S2:** Technical replicates and linear instrument response analysis of the mass spectrometer.
**Figure S3:** Technical replicates of LC–MS analysis skinned human single muscle fibers (hSMFs) demonstrate high reproducibility.
**Figure S4:** Reproducibility of hSMF inter‐donor biological replicates.
**Figure S5:** Top–down LC–MS of fast isoform myosin light chain 3 in hSMFs from different donors.
**Figure S6:** Top–down LC–MS of actin isoforms in hSMFs from different donors.

## Data Availability

The data that support the findings of this study are openly available in MassIVE at https://massive.ucsd.edu/ProteoSAFe/static/massive.jsp, reference number MSV000100493.
