# Supplementary material for: Top–Down Proteomics of Skinned Human Muscle Fibers Reveals Proteoform‐Resolved Fiber‐to‐Fiber Variability
Source: J Mass Spectrom. 2026 Feb 22;61(3):e70040. doi: 10.1002/jms.70040 (PMC12926692; doi:10.1002/jms.70040)
Supplement: Supplementary file 1 — Table S1: Summary of identified skeletal sarcomere proteins. Figure S1: Method optimization of surfactant‐free extraction of proteins from skinned human single muscle fibers (hSMFs). Figure S2: Technical replicates and linear instrument response analysis of the mass spectrometer. Figure S3: Technical replicates of LC–MS analysis skinned human single muscle fibers (hSMFs) demonstrate high reproducibility. Figure S4: Reproducibility of hSMF inter‐donor biological replicates. Figure S5: Top–down LC–MS of fast isoform myosin light chain 3 in hSMFs from different donors. Figure S6: Top–down LC–MS of actin isoforms in hSMFs from different donors. [file JMS-61-e70040-s001.docx]

***Supporting Information***

**Top-down Proteomics of Skinned Human Muscle Fibers Reveals Proteoform-Resolved Fiber-to-Fiber Variability**

Mallory C. Wilson^1,2^, Zhan Gao^2^, Justin R. Lopez^3^, Yanlong Zhu^4^, Szczepan S. Olszewski^5^, Adam R. Konopka^5,6^, Gary M. Diffee^3^, and Ying Ge^1,2,4^*

^1^ Department of Chemistry, University of Wisconsin-Madison, Madison, WI 53706, USA

^2^ Department of Cell and Regenerative Biology, University of Wisconsin-Madison, Madison, WI 53705, USA

^3^Department of Kinesiology, University of Wisconsin-Madison, Madison, WI 53706, USA

^4^Human Proteomics Program, School of Medicine and Public Health, University of Wisconsin-Madison, Madison, WI 53705, USA

^5^Division of Geriatrics and Gerontology, Department of Medicine, University of Wisconsin-Madison, Madison, WI 53705, USA

^6^Wisconsin Nathan Shock Center of Excellence in the Basic Biology of Aging, University of Wisconsin-Madison, Madison, WI 53705, USA

*Correspondence: Dr. Ying Ge: [ying.ge@wisc.edu](mailto:ying.ge@wisc.edu)

**Table of Contents**

***Supplementary Tables***

Supplementary Table 1. Summary of identified skeletal sarcomere proteins .................S-3

***Supplementary Figures***

Supplementary Figure S1. Method optimization of surfactant-free extraction of proteins from skinned human single muscle fibers (hSMFs) ........................................................S-4

Supplementary Figure S2. Technical replicates and linear instrument response analysis of the mass spectrometer .....................................................................................................S-5

Supplementary Figure S3. Technical replicates of LC-MS analysis skinned human single muscle fibers (hSMFs) demonstrate high reproducibility ...............................................S-6

Supplementary Figure S4. Reproducibility of hSMF inter-donor biological

replicates ..........................................................................................................................S-7

Supplementary Figure S5. Top-down LC-MS of fast isoform myosin light chain 3 in hSMFs from different donors ..........................................................................................S-8

Supplementary Figure S6. Top-down LC-MS of actin isoforms in hSMFs from different donors ..............................................................................................................................S-9

References .................................................................................................................................S-10

***Supplementary Tables***

**Table S1. Summary of identified skeletal sarcomere proteins.** Retention time (RT), gene name, proteoform name, experimental monoisotopic mass (M_r_ Exp’t), calculated monoisotopic mass (M_r_ Calc’d), and sequence modifications for the proteoforms identified in this study. Proteins were identified at the MS1 level based on accurate molecular weight measurements and previously published data. Abbreviations: acetylation (Acetyl), phosphorylation (Phospho), methionine removal (-Met), cysteine removal (-Cys), methylation (Methyl), hydroxyproline (Hydropro), amino acids (AA).

| ***RT (min)*** | ***Gene*** | ***UniProt ID*** | ***M_r_ Calc’d (Da)*** | ***M_r_ Exp't (Da)*** | ***Error (ppm)*** | ***Proteoform*** | ***PTMs*** | ***Extracted ions for expression quantification (+/- 0.2 m/z)*** |
| --- | --- | --- | --- | --- | --- | --- | --- | --- |
| ~20 | TNNI1 | P19237 | 21560.6 | 21560.4 | 10.8 | *ss*TnI | -Met | 674.7685; 696.5377; 719.6835; 744.5035; 771.0215; 799.5026 |
| ~20.5 | TNNI2 | P48788 | 21248.9 | 21248.7 | 10.6 | *fs*TnI | -Met, Acetyl | 709.2987; 733.6874; 759.8904; 787.9976; 818.2278 |
| ~30 | MYL3 | P08590 | 21841.9 | 21841.8 | 6.1 | MLC1V | -Met, Trimethyl | 754.2073; 781.1074; 809.9627; 841.0766; 874.6792; 911.0817 |
| ~30.5 | TPM1 | P09493 | 32749.8 | 32749.4 | 13.7 | αTpm | Acetyl | 745.3358; 762.6147; 780.7524; 799.7685; 819.7691; 840.744 |
|  | TPM2 | P07951 | 32892.6 | 32892.3 | 9.1 | βTpm | Acetyl | 748.5515; 765.9276; 784.1404; 803.2597; 823.2981; 844.3843 |
| ~32 | MYL1 | P05976 | 21055.8 | 21055.6 | 9.5 | MLC1F | -Met, Acetyl | 780.8454; 810.8392; 843.1922; 878.2834; 916.4692; 958.0355 |
| ~32.5 | MYL11 | Q96A32 | 18925.4 | 18925.2 | 9.2 | MLC2F | -Met, Trimethyl | 789.5198; 823.8031; 861.2029; 902.1646; 947.2222; 997.0232 |
| ~34.5 | MYL1 | P05976-02 | 16594.2 | 16594.0 | 10.5 | MLC3F | -Met, Acetyl | 922.8449; 977.1299; 1038.0746; 1107.2793; 1186.2979; 1277.3992 |
| ~35 | MYL2 | P10916 | 18699.4 | 18699.3 | 7.1 | MLC2S | -Met, Trimethyl | 814.0177; 850.9738; 891.4950; 936.0182; 985.1774 |
| ~41 | TNNC2 | P02585 | 18032.4 | 18032.1 | 15.3 | *fs*TnC | -Met, Acetyl | 950.0723; 1002.7972; 1061.7266; 1128.0210; 1023.1543; 1289.0940 |
| ~41.5 | ACTA1 | P68133 | 41871.9 | 41871.5 | 10.3 | sα-actin | -Met, -Cys, Acetyl, Me | 822.0201; 838.4358; 855.5117; 873.3310; 891.9037; 911.2375 |
| ~42 | TNNC1 | P63316 | 18443.6 | 18443.4 | 9.0 | *ss*TnC | -Met, Acetyl | 971.7198; 1025.7032; 1085.8615; 1153.7278; 1230.6424 |

***Supplementary Figures***

**
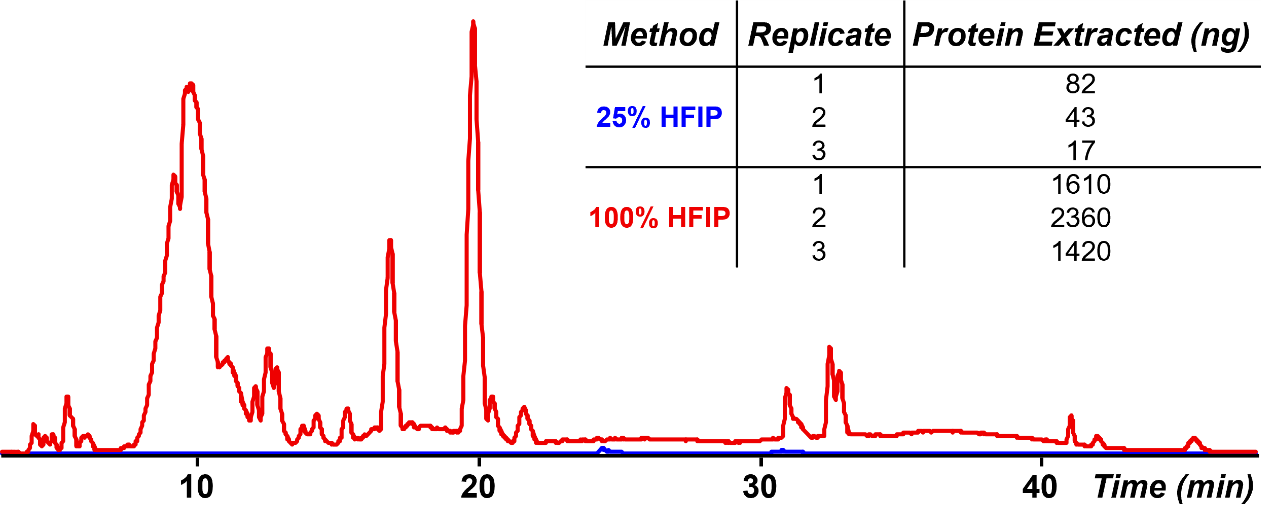
**

**Figure S1. Method optimization of surfactant-free extraction of proteins from skinned human single muscle fibers (hSMFs).** Representative BPC traces of extracted proteins from an hSMF using the original 25% HFIP extraction method^1^ (blue) and the optimized 100% extraction method (red). The table on the top right shows the total protein extracted from each sample in a triplicate of treated hSMFs. These results indicate that increasing the concentration of HFIP in the extraction buffer results in consistently successful sarcomere extractions.

**
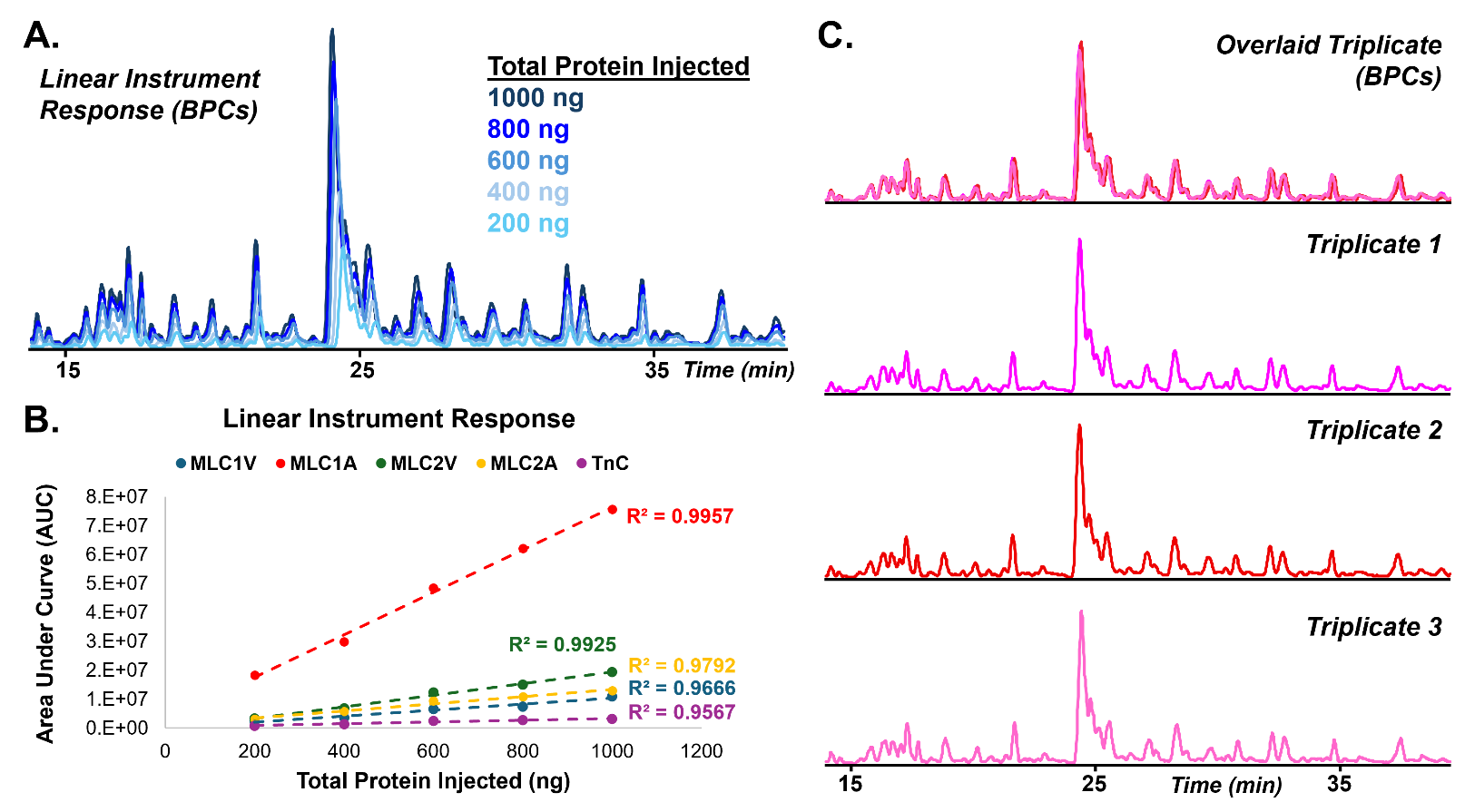
****Figure S2. Technical replicates and linear instrument response analysis of the mass spectrometer. A)** Base peak chromatograms (BPCs) of variable total protein are injected to identify the linear response range of the mass spectrometer using a standard sarcomere protein extract.^2^ **B)** Elution times of select sarcomere proteins identified the most abundant 5-7 charge state ions from the non-deconvoluted spectra, which were used to produce extracted ion chromatograms (EICs) across different total protein injections. Area under the curve of each EIC was plotted as a function of total protein loaded for each selected protein. **C)** Three technical (injection, 400 ng) replicates as an assessment of the stability of the mass spectrometer. The triplicate overlay of the BPCs reveals the high similarity between traces, indicating that the instrument’s response can be considered reproducible during data collection.

**
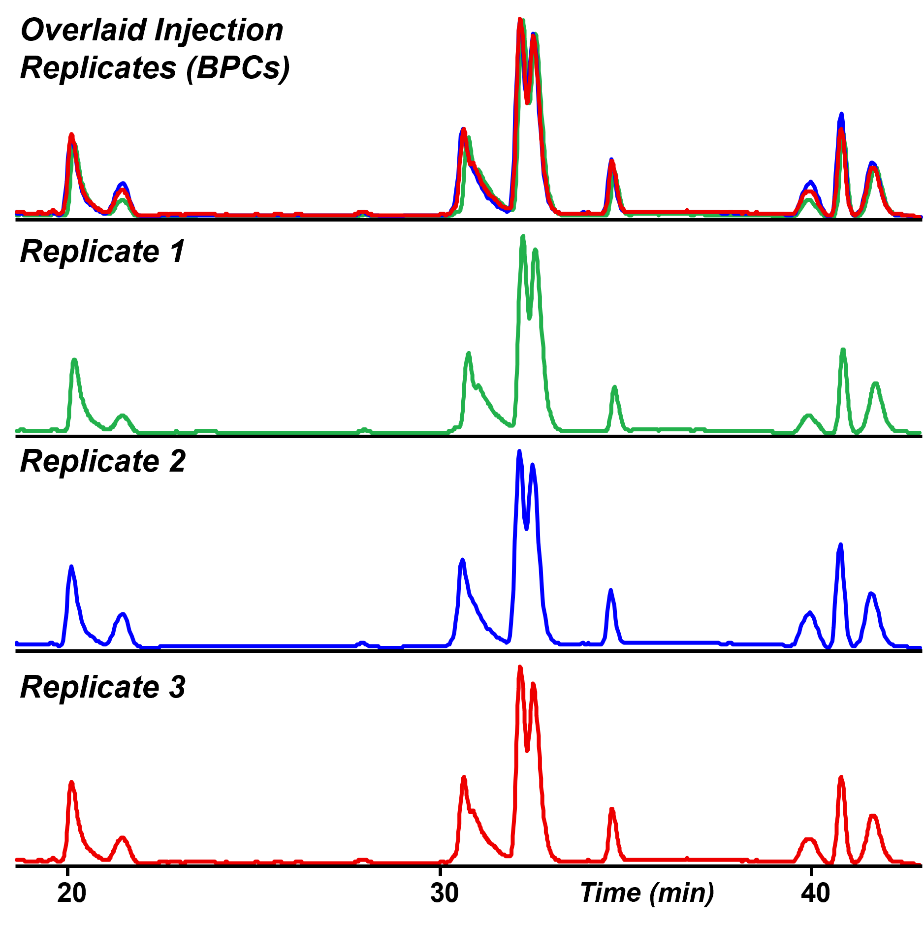
**

**Figure S3. Technical replicates of LC-MS analysis skinned human single muscle fibers (hSMFs) demonstrate high reproducibility.** Three technical (injection, 400 ng) replicates as an assessment of the stability of the mass spectrometer. The triplicate overlay of the BPCs reveals the high similarity between traces, indicating that the instrument’s response can be considered reproducible for the skinned human single muscle fiber (hSMF) injections.


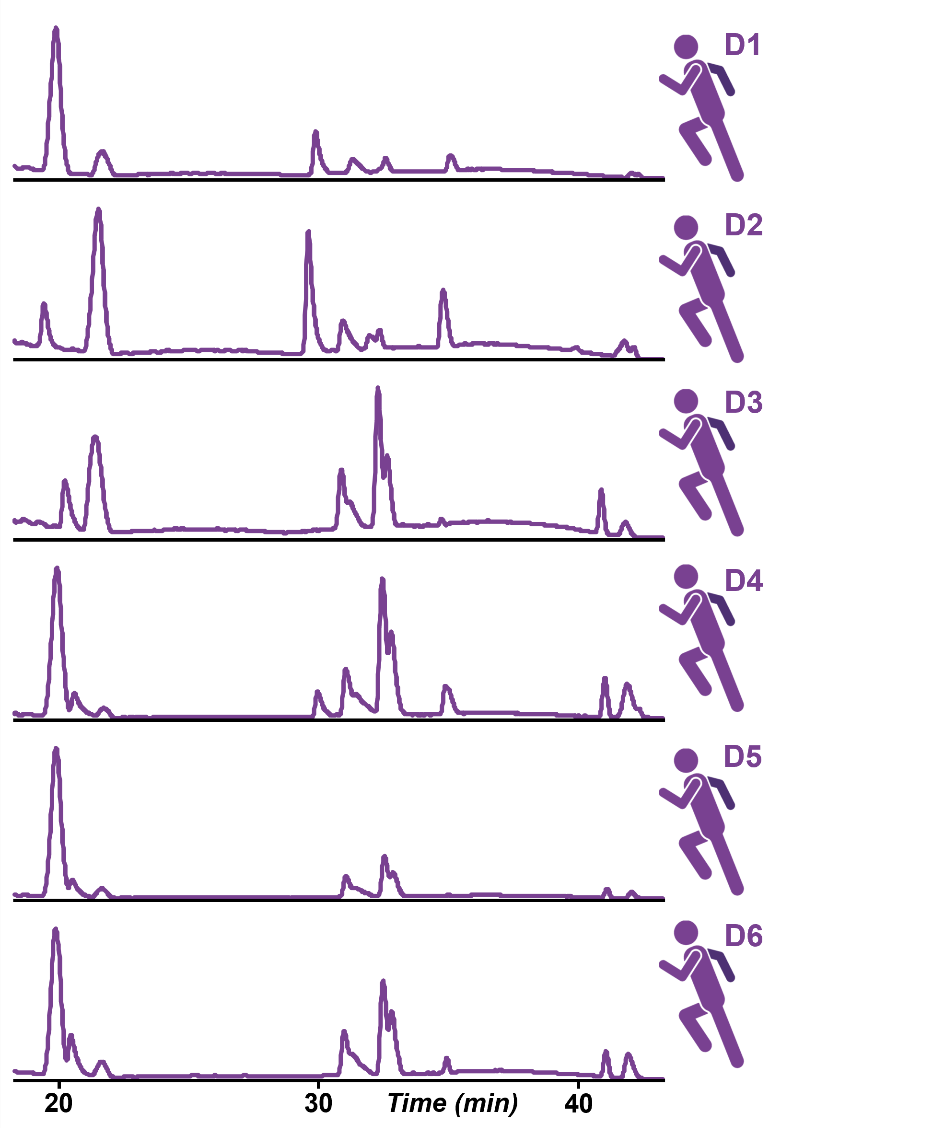


**Figure S4.** **Reproducibility of hSMF inter-donor biological replicates.** BPCs of each biological replicate exhibit high reproducibility of elution times despite each fiber’s unique isoform expression.

**
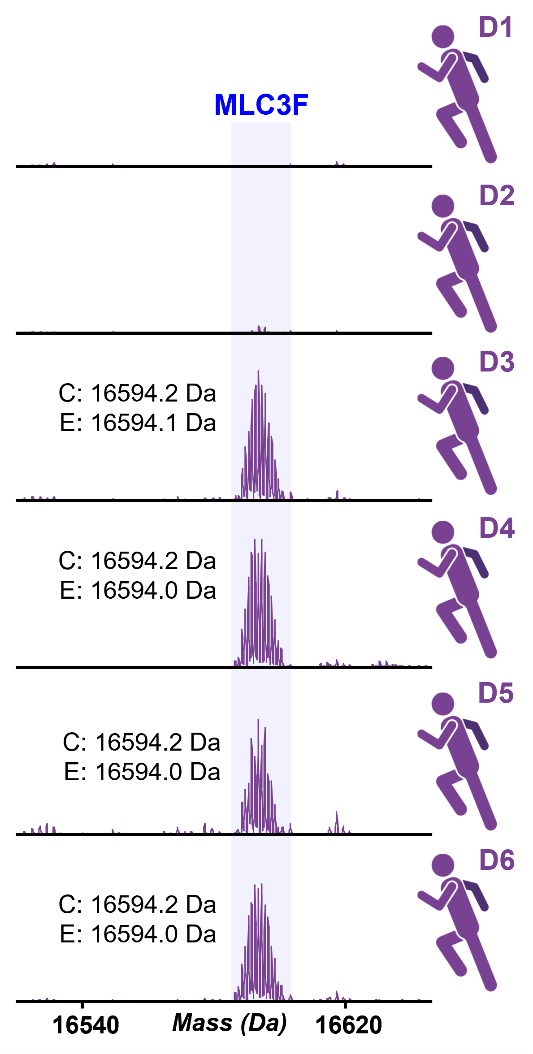
**

**Figure S5.** **Top-down LC-MS of fast isoform myosin light chain 3 in hSMFs from different donors.** Deconvoluted spectra of fast isoform MLC3F in each sample across cohorts. Calculated monoisotopic mass (C) and experimental monoisotopic mass (E) are displayed beside each peak. MLC3F was not detected in samples D1 or D2.

**
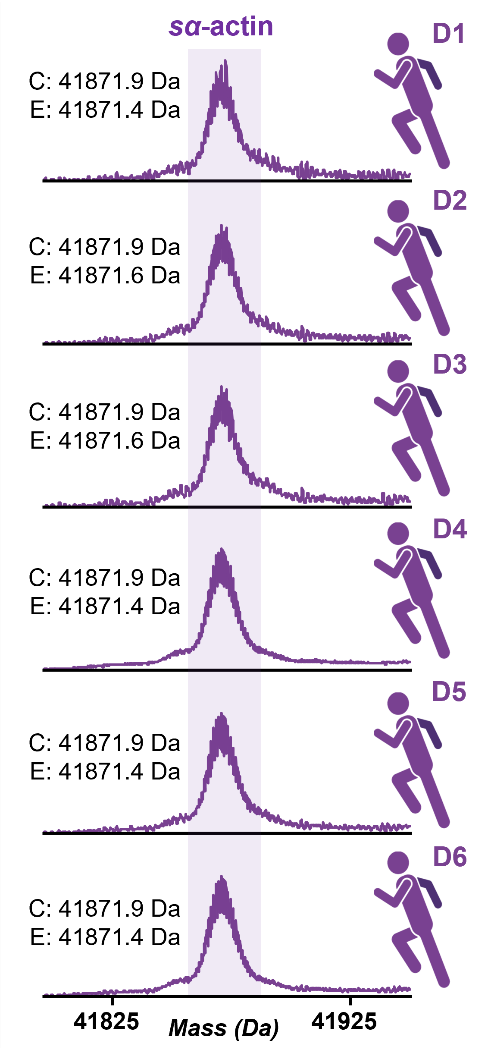
**

**Figure S6. Top-down LC-MS of actin isoforms in hSMFs from different donors.** Deconvoluted spectra of fast isoform sα-actin in each sample across cohorts. Calculated monoisotopic mass (C) and experimental monoisotopic mass (E) are displayed beside each peak. sα-actin was detected in all samples.

***References***

(1) Melby, J. A.; Brown, K. A.; Gregorich, Z. R.; Roberts, D. S.; Chapman, E. A.; Ehlers, L. E.; Gao, Z.; Larson, E. J.; Jin, Y.; Lopez, J. R.; Hartung, J.; Zhu, Y.; McIlwain, S. J.; Wang, D.; Guo, W.; Diffee, G. M.; Ge, Y. High Sensitivity Top–down Proteomics Captures Single Muscle Cell Heterogeneity in Large Proteoforms. *Proceedings of the National Academy of Sciences* 2023, *120* (19), e2222081120. https://doi.org/10.1073/pnas.2222081120.

(2) Wilson, M. C.; Josvai, M.; Walters, J. K.; Lawson, J.; Rossler, K. J.; Gao, Z.; Zhu, Y.; Kamp, T. J.; Crone, W. C.; Eckhardt, L. L.; Ge, Y. High-Sensitivity Top-Down Proteomics Reveals Enhanced Maturation of Micropatterned Induced Pluripotent Stem Cell-Derived Cardiomyocytes. *J. Proteome Res.* 2025, *24* (9), 4335–4343. https://doi.org/10.1021/acs.jproteome.5c00505.
